# Seismic Response and Fragility Analysis of a Single-Pylon Cable-Stayed Bridge Considering the Construction Process

**DOI:** 10.3390/ma19183948

**Published:** 2026-09-17

**Authors:** Xiangliang Tang, Pan Mao, Guanghui Liu, Haodong Xu, Huaxu Zhang, Yudong Zhang, Xiaoxian Liu, Zengliang Liu

**Affiliations:** 1 Fuyang Transportation Energy Investment Group Co., Ltd., Fuyang 236000, China; 13940325122@163.com (X.T.); 13457505160@163.com (G.L.); 15655567437@163.com (H.X.); 2 Anhui Transport Consulting and Design Institute Co., Ltd., Hefei 230088, China; 15256953852@163.com; 3 College of Civil Engineering, Hefei University of Technology, Hefei 230009, China; playerseb@outlook.com (Y.Z.); jiangsulxx@163.com (X.L.); 2025110726@mail.hfut.edu.cn (Z.L.)

**Keywords:** single-pylon cable-stayed bridge, construction stage, pylon–girder rigid-connection system, semi-floating system, seismic fragility analysis

## Abstract

The structural system, construction stage, and damper parameters have significant influences on the seismic performance of single-pylon cable-stayed bridges. To investigate the seismic responses of the bridge under various construction stages, nonlinear finite-element models were established in OpenSees for the pylon stage, large cantilever stage and completed bridge stage. Parametric analysis was carried out to investigate the effect of pylon-to-girder connection and viscous dampers. Incremental dynamic analysis, parametric analysis and fragility analysis were carried out with the finite element model. The results indicate that, compared with the semi-floating system, the pylon–girder rigid-connection system provides stronger longitudinal restraint, leading to smaller pylon-top displacement, girder displacement, and bearing displacements, but results in a higher bending moment and curvature demand at the pylon base. Installing viscous dampers in the longitudinal direction can reduce bearing displacements. The probabilities of girder unseating damage and pylon-base bending failure continuously increase as construction progresses from the pylon stage to the large cantilever stage and finally to the completed-bridge stage. The fragility analysis further shows that the rigid-connection and semi-floating systems exhibit different vulnerability characteristics at both the component and system levels.

## 1. Introduction

Earthquakes are among the most common natural hazards, characterized by high aleatory variability and limited predictability [1]. In recent decades, earthquake-induced losses have escalated worldwide. Representative examples include the 2008 Wenchuan Earthquake, the 2017 Jiuzhaigou Earthquake, as well as the 2010 Canterbury Earthquakes and the 2011 Great East Japan (Tohoku) Earthquake. Although cable-stayed bridges belong to the category of long-span flexible bridges, they are still vulnerable to seismic damage. Historical seismic cases show that severe flexural failure occurred at the base of the main pylon of the under-construction Jilu Bridge during the 1999 Chi-Chi earthquake in Taiwan [2]. Damage was also observed on the unfinished Miaoziping Min River Bridge in the 2008 Wenchuan earthquake [3].

In terms of the seismic response research of cable-stayed bridges, Liu et al. [4] conducted seismic fragility analysis of a completed single-pylon highway cable-stayed bridge with double cable planes using the incremental dynamic analysis (IDA) method. The results revealed that bearings are the most vulnerable components of the bridge. Pylon damage is mainly concentrated at the pylon base, and the damage probability of the overall bridge system is higher than that of any individual structural component. Zhang et al. [5] performed asynchronous multi-support excitation analyses of the completed Cable-Stayed Bridge through three-dimensional nonlinear finite element time-history analysis considering both geometric and material nonlinearities. The results indicated that the wave-passage effect significantly alters the seismic response amplitudes of pylons, girders, and bearings, and neglecting spatial ground motion variations can underestimate the seismic demands of bridge components. Bayraktar et al. [6] investigated the seismic responses of a cable-stayed bridge at the large cantilever construction stage under strong earthquakes using nonlinear finite element time-history analysis. The findings demonstrated that remarkable amplification effects occur in girder displacement, pylon-base bending moment, and stay cable tension, even if no visible structural damage is observed in the large cantilever state. Meanwhile, Wilson J C [7], Benjumea J [8] and other scholars verified the viewpoint that bridges under construction possess poorer seismic capacity and are more vulnerable to earthquake-induced damage. Currently, most existing studies on the seismic performance of cable-stayed bridges primarily focus on the in-service completed-bridge stage, while limited research has focused on the mechanical responses and seismic fragility of cable-stayed bridges during construction.

In terms of seismic mitigation research on cable-stayed bridges, Ren et al. [9] conducted shake-table tests on a 1/20 scaled model to investigate the seismic control performance of viscous dampers installed on a single-pylon cable-stayed bridge subjected to near-field ground motions. The test results verified that viscous dampers can effectively restrain the longitudinal displacement of the bridge girder. However, near-field pulse-type seismic excitations significantly increase the deformation and force demands of dampers. Barnawi et al. [10] adopted the IDA method to evaluate the seismic fragility of cable-stayed bridges equipped with viscous dampers and other seismic mitigation devices. The results indicated that such devices can effectively reduce the damage exceedance probabilities of pylons and bearings, whereas their mitigation efficiency is constrained by the spectral characteristics and intensity of ground motions. Overall, most existing studies focus on the damping effectiveness of viscous dampers for cable-stayed bridges in the completed in-service state, while investigations on their seismic mitigation performance throughout different construction stages remain insufficient.

Motivated by these research gaps, this paper investigates the seismic performance and fragility of a single-pylon cable-stayed bridge under construction. The specific objectives of this study are to (i) investigate the seismic response characteristics of bridges with different structural systems; (ii) investigate the seismic-response law of single-pylon double-leg cable-stayed bridges under different construction stages; (iii) investigate the seismic mitigation effect of viscous dampers under different construction stages; and (iv) by study the damage probability of bridges under seismic excitation, delivering supporting evidence for catastrophe risk financing in construction projects, so as to mitigate potential losses of public and private assets.

## 2. Prototype Bridge

**Figure 1:**
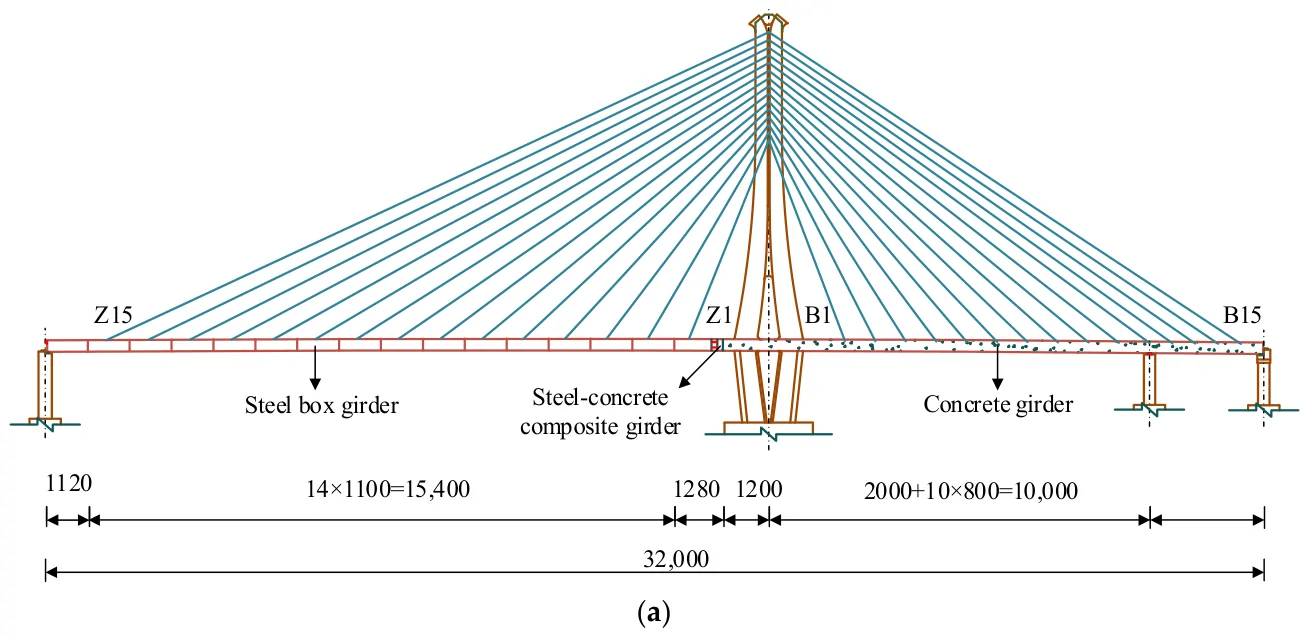

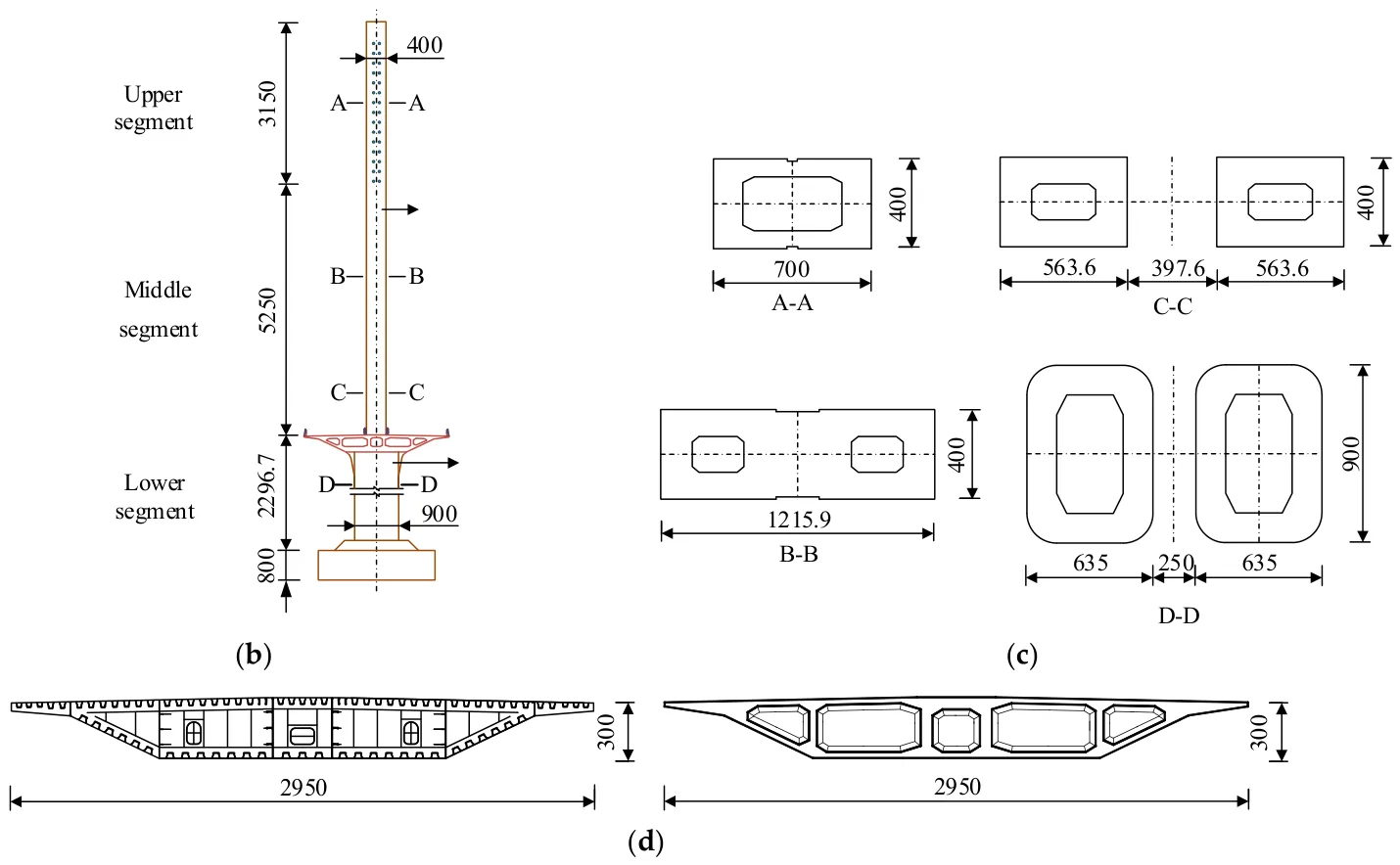
Configuration of the single-pylon cable-stayed bridge (unit: cm): (**a**) elevation view; (**b**) general arrangement of the pylon (Sections A–D are reference sections for numerical modelling.); (**c**) cross-sections of pylon at different heights; (**d**) steel box-girder and concrete box-girder cross-sections.

This study adopts a single-pylon cable-stayed bridge with a composite girder as the prototype. The bridge adopts an integral deck configuration with a standard deck width of 29.5 m. The main span is 190 m and employs a steel box girder. The side span is 130 m and utilizes a cast-in-place C50 concrete box girder. An auxiliary pier is arranged in the side span. A 12.8 m long steel–concrete hybrid transition segment is located near the pylon. The pylon is cast in place with C50 concrete and has a total height of 106.967 m. As shown in Figure 1a,b, the pylon has a vase-shaped profile in the longitudinal direction and is divided from top to bottom into a 31.5 m upper segment, a 52.5 m middle segment, and a 22.967 m lower segment. The upper segment has a constant cross-section with a single-cell single-box configuration (Section A-A in Figure 1c), measuring 7 m in the longitudinal direction and 4 m in the transverse direction. The middle segment transitions from a single-cell single-box to a single-box double-cell, and finally to a double-box single-cell configuration (Sections A-A, B-B, and C-C in Figure 1c). Throughout this segment, the transverse width remains 4 m, while the longitudinal width increases from 7.0 m to 18.0 m; at the pylon–girder junction, the clear spacing between the two boxes is 6.0 m. The lower segment adopts a double-box single-cell section (Section D-D in Figure 1c). At its intersection with the bottom of the main girder, the clear spacing between the two boxes is 6.0 m, matching the 6.0 m clear spacing at the pylon–girder junction of the middle segment; toward the pile–cap interface, this spacing reduces to 1.0 m.

Both the main and side spans are equipped with 15 pairs of stay cables. Standard cable spacing is 11.0 m along the main-span steel box girder, 8.0 m along the side-span concrete box girder, and 6.0 m between the auxiliary pier and the transition pier. The stay cables consist of epoxy-coated, PE-sheathed prestressing steel strands with a nominal tensile strength of 1860 MPa. Specifically, main-span cables Z1–Z6 and side-span cables B1–B4 use 15.2–34 strands with a total steel area of 4726 mm^2^; main-span cables Z7–Z8 and side-span cables B5–B6 use 15.2–37 strands with a total steel area of 5143 mm^2^; and main-span cables Z9–Z15 and side-span cables B7–B15 use 12.5–43 strands with a total steel area of 5977 mm^2^.

## 3. Finite Element Model

### 3.1. Research Methodology

**Figure 2:**
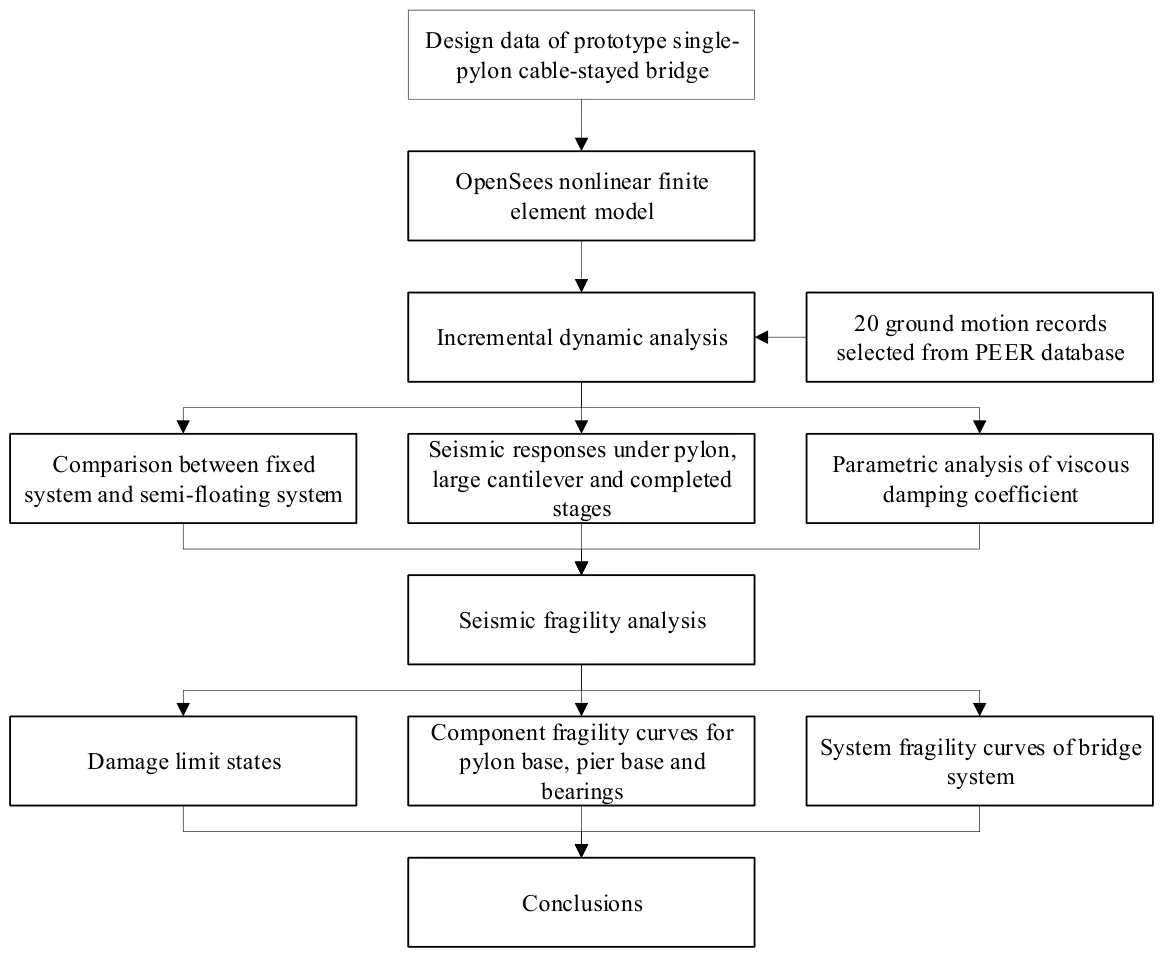
Flowchart of research methodology.

The research methodology adopted in this study proceeds as follows. First, relevant bridge parameters are collected. A refined nonlinear finite element model of the bridge is then established using OpenSees 3.3.0, with comparative cases designed to cover different pylon–girder connection configurations and construction stages. Next, appropriate ground motion records are selected for subsequent simulations. Incremental dynamic analysis (IDA) is implemented to perform nonlinear time-history analysis across all cases, followed by seismic fragility analysis at both component and system levels. Ultimately, the seismic response and damage evolution laws of the target bridge under various structural systems and construction stages are obtained. The detailed methodological procedure is illustrated in Figure 2.

### 3.2. Model Parameters

**Figure 3:**
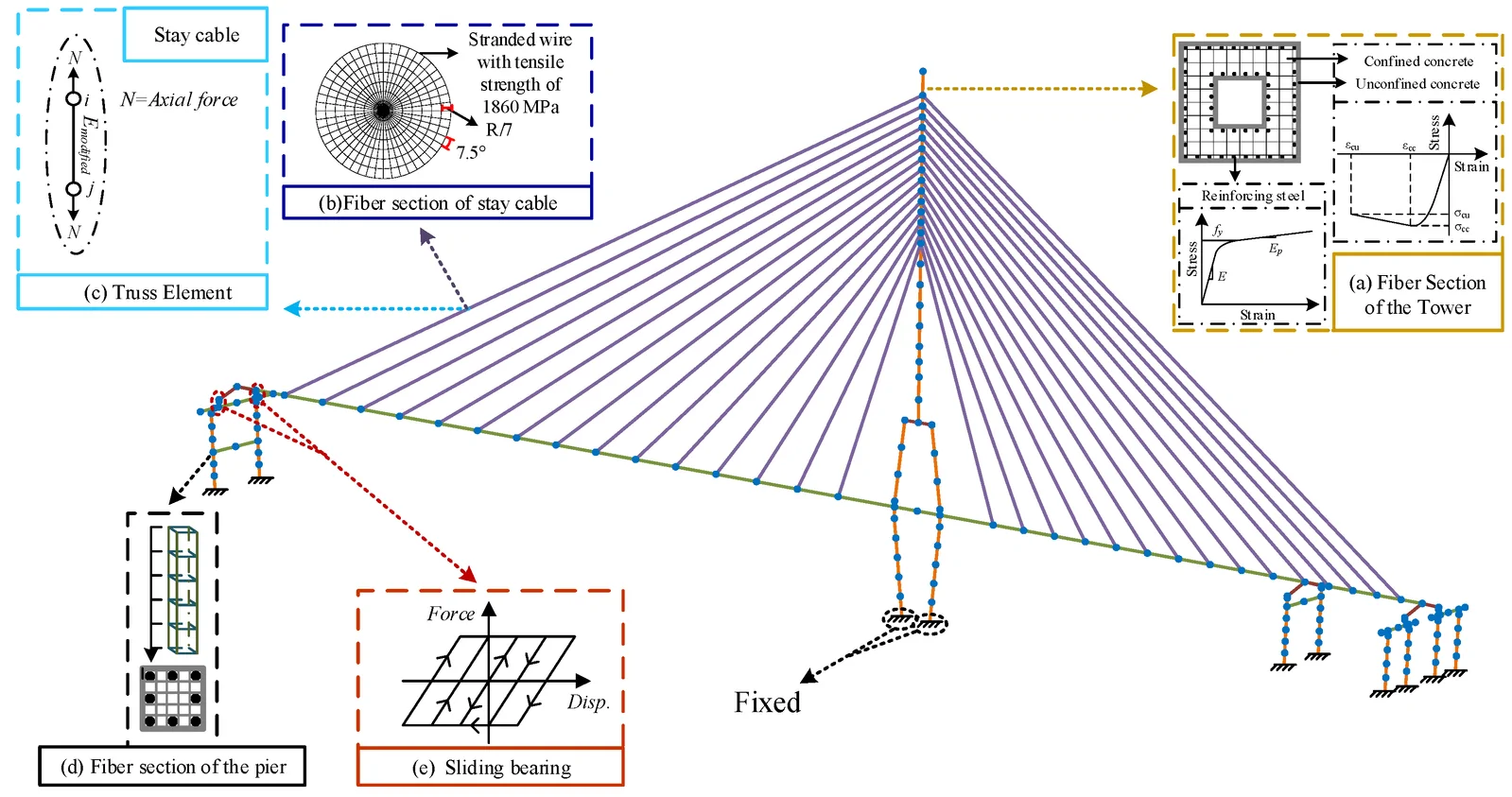
Finite element model of the cable-stayed bridge: (**a**) fiber section of the pylon; (**b**) fiber section of stay cable; (**c**) truss element; (**d**) fiber section of the pier; (**e**) sliding bearing.

A nonlinear finite element (FE) model of the cable-stayed bridge was developed in OpenSees [11,12], and the general configuration of the numerical model is shown in Figure 3. The pylon, auxiliary piers, and transition piers were modeled using force-based beam–column elements, which capture the spread of plasticity along the member length. A bilinear elastoplastic constitutive model is adopted to simulate the mechanical behavior along the longitudinal bridge direction. The critical sliding force of the bearing is determined by multiplying the vertical reaction force of the bearing by the friction coefficient μ, where the friction coefficient μ of the sliding bearing is set to 0.02. From the pylon construction stage to the completed bridge stage, the critical sliding force of bearings at the pylon–girder connection of the semi-floating system increases from 551 kN to 1095 kN. The numerical model of the fixed pylon–girder system in this study is established based on the design documents of the prototype bridge, with identical pylon–girder connections to the real bridge. Accordingly, its simulation results can better reflect the actual mechanical behavior of the engineering structure, and this model is taken as the benchmark calculation model of the present research. As a cable-stayed bridge system more suitable for strong earthquake zones, the semi-floating system is only set as a comparative working condition to investigate the effect of pylon–girder connection on the seismic response, damage evolution and fragility curves of the bridge.

To represent the variable cross-section of the pylon, four representative sectional configurations were defined along its height. Each sectional configuration was assigned to multiple force-based beam–column elements within the corresponding height interval, resulting in a total of 34 elements for the pylon. This approach allows efficient modeling of the sectional variation while simultaneously accounting for nonlinear constitutive behavior and axial force–bending moment interaction. Along each force-based beam–column element, five equally spaced integration points were assigned fiber sections comprising confined (core) concrete fibers, unconfined (cover) concrete fibers, and mild steel fibers [13,14,15]. The girder elements are divided according to cable anchoring positions and construction joints. A Newmark constant average acceleration method was used as an analysis algorithm. Lumped mass distribution is employed to model the entire bridge structure. The time step of each ground motion record is directly used as the analysis time increment.

The Concrete02 constitutive material model in OpenSees is adopted to simulate concrete in this study. The compressive backbone curve follows the confined concrete theory of Kent–Park modified by Scott et al. (1982) [16]. The peak compressive strength, peak strain and ultimate compressive strain of confined concrete are quantitatively calculated using the complete formulas of this model.

The enhanced peak compressive strength *f_cc_* of concrete accounting for lateral confinement is calculated via Equation (1) [17].

(1) $$f_{cc}=f_{c}\left(-1.254+2.254\sqrt{1+\frac{7.94f_{l}}{f_{c}}}-2\frac{f_{l}}{f_{c}}\right)$$

where *f_c_* denotes the axial compressive strength of unconfined concrete; and *f_l_* denotes the equivalent lateral confinement stress.

The ultimate compressive strain of confined concrete is calculated by Equation (2).

(2) $$\varepsilon_{cu}=0.004+1.4\frac{\rho_{s}f_{g}\varepsilon_{su}}{f_{cc}}$$

where *ε_su_* denotes the ultimate tensile strain of transverse reinforcement; *ρ_s_* denotes the transverse reinforcement ratio; and *f_g_* denotes the yield strength of transverse reinforcement.

Material parameters for confined concrete were the pylon peak compressive strength *f_cc_* = 40,525 kPa with ultimate strain *ε_cu_* = 0.0124; transition pier peak compressive strength f*_c_* = 38,631 kPa with *ε_cu_* = 0.0104; and auxiliary pier peak compressive strength *f_cc_* = 38,256 kPa with *ε_cu_* = 0.0101. For the constitutive modeling of reinforcing steel, the Steel02 material model implemented on the modified Menegotto–Pinto framework is adopted for longitudinal reinforcement to capture the Bauschinger effect, and the hardening ratio is 0.005 [18], with an initial Young’s modulus of 200 GPa and a yield strength of 400 MPa.

Stay cables were modeled using truss elements, and the pretension was introduced by prescribing initial stress to the cable material. The main girder was assumed to remain elastic under the considered earthquake excitations and was therefore modeled with elastic beam–column elements. The cap beams and struts at the auxiliary and transition piers, which may enter the inelastic range during strong ground motion, were also modeled using force-based beam–column elements. The pylon-to-girder connection was taken as rigid. The bearings at the auxiliary and transition piers were modeled as ideal elastic-perfectly plastic friction elements with a friction coefficient of 0.02.

Geometric nonlinearity and P-Δ second-order effects are incorporated into this study. The cable sag effect is captured via Ernst’s formula. Variations in cable force at different construction stages are accounted for to reflect their influence on cable stiffness. Cable forces are extracted from the Midas model with full construction sequences implemented, such that cable tension loss is fully considered. The cable forces in the completed bridge stage range from 3562 kN to 8442 kN.

The vertical reaction forces at the pylon base of the OpenSees numerical model and Midas design model are 258,418 kN and 264,104 kN, respectively. The discrepancy between the two sets of results is within an acceptable margin, demonstrating that the established OpenSees model can accurately reflect the actual structural behavior for subsequent numerical analyses.

## 4. Input Ground Motions

**Figure 4:**
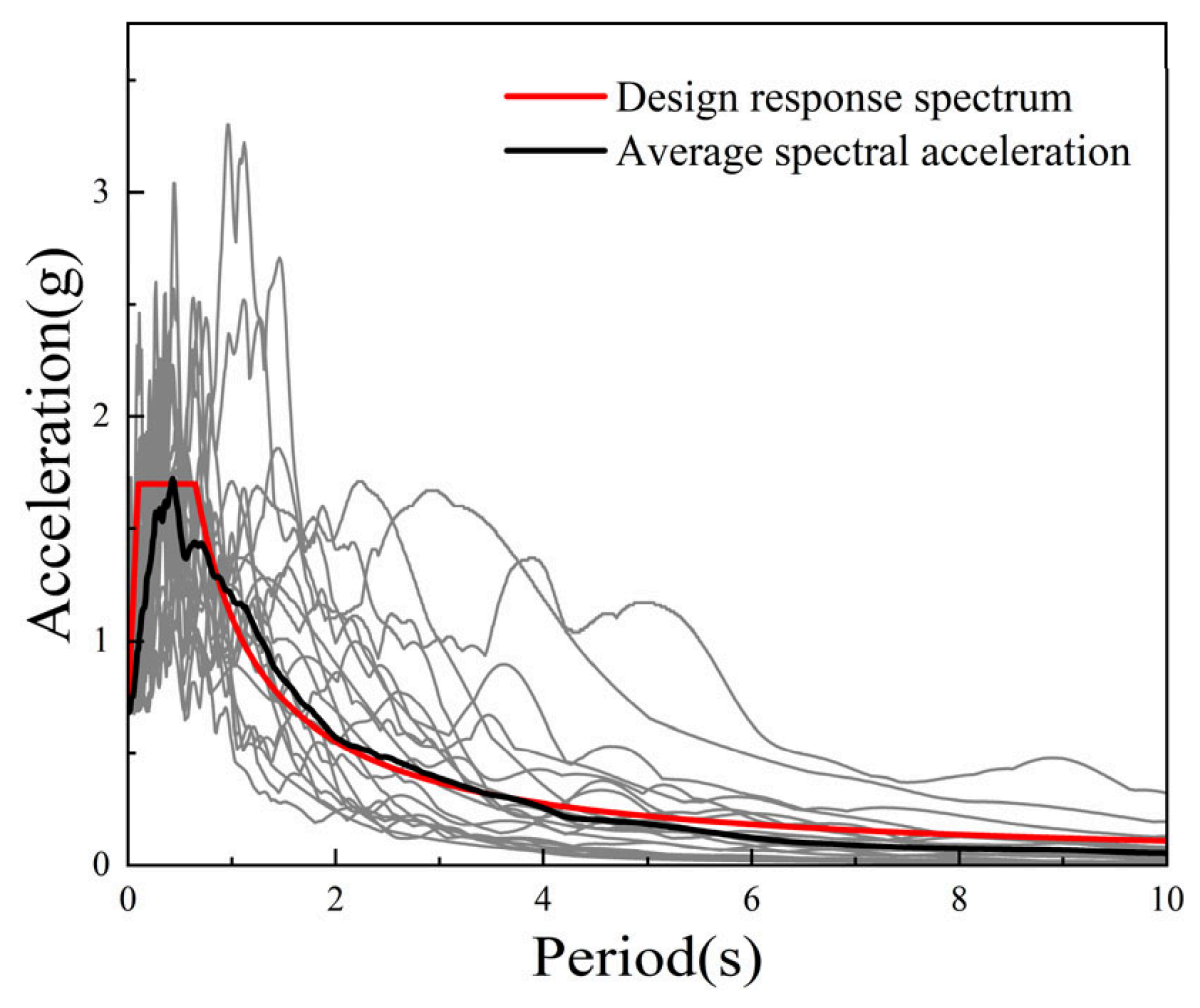
The target response spectrum and the response spectra of ground motions.

To explore the seismic response of the cable-stayed bridge, a total of 20 ground motion records were selected from the strong-motion database of the Pacific Earthquake Engineering Research (PEER) Center [19]. All records were taken from the horizontal H1 component, and the detailed characteristics of the selected ground motion records are summarized in Table 1. The selected records have moment magnitudes ranging from approximately 5.6 to 7.36 and epicentral distances ranging from 15 to 46 km, representative of near-field to intermediate-field ground motions [20,21]. In addition, the inherent record-to-record variability required for the development of probabilistic seismic demand models is preserved. The unscaled peak ground accelerations of these recorded motions range from approximately 0.079 g to 0.236 g, ensuring realistic baseline seismic intensity prior to amplitude scaling. The bridge site is categorized as Class IV soil with a design peak ground acceleration (PGA) of 0.4 g. The corresponding peak ground acceleration under the strong earthquake (E2) action is 0.68 g. The site has favorable geological conditions and is free of adjacent active faults. Only the horizontal H1 component is adopted in the nonlinear time-history analyses. The target response spectrum generated according to the Chinese code was input into the PEER database, where spectrum-matching was performed to obtain corresponding ground-motion records. The Minimize MSE algorithm was used for spectral matching. The period range for comparison against the target response spectrum was 0.1 s–1.34 s. The target response spectrum and the response spectra of the 20 ground motions are shown in Figure 4. At a PGA of 0.68 g, the scaling coefficients ranged from 2.9 to 11.5, since ground-motion records were scaled by peak ground acceleration (PGA) for IDAs. Nonlinear time-history analyses were performed along the longitudinal direction of the bridge. The fundamental longitudinal vibration period and the period corresponding to 90% effective mass participation in the longitudinal direction were taken as two characteristic periods to solve for Rayleigh damping coefficients, and a damping ratio of 3% was specified in all numerical cases. Current stiffness was adopted throughout the nonlinear analysis process [22].

The site belongs to Site Class IV. Only the horizontal H1 component is adopted in the nonlinear time-history analyses. The target response spectrum generated according to the Chinese code was input into the PEER database, where spectrum-matching was performed to obtain corresponding ground-motion records. The Minimize MSE algorithm was used for spectral matching. The period range for comparison against the target response spectrum was 0.1 s–1.34 s. At a PGA of 0.68 g, the scaling coefficients ranged from 2.9 to 11.5, since ground-motion records were scaled by peak ground acceleration (PGA) for IDAs.

## 5. Seismic Response of Different Structural Systems

**Figure 5:**
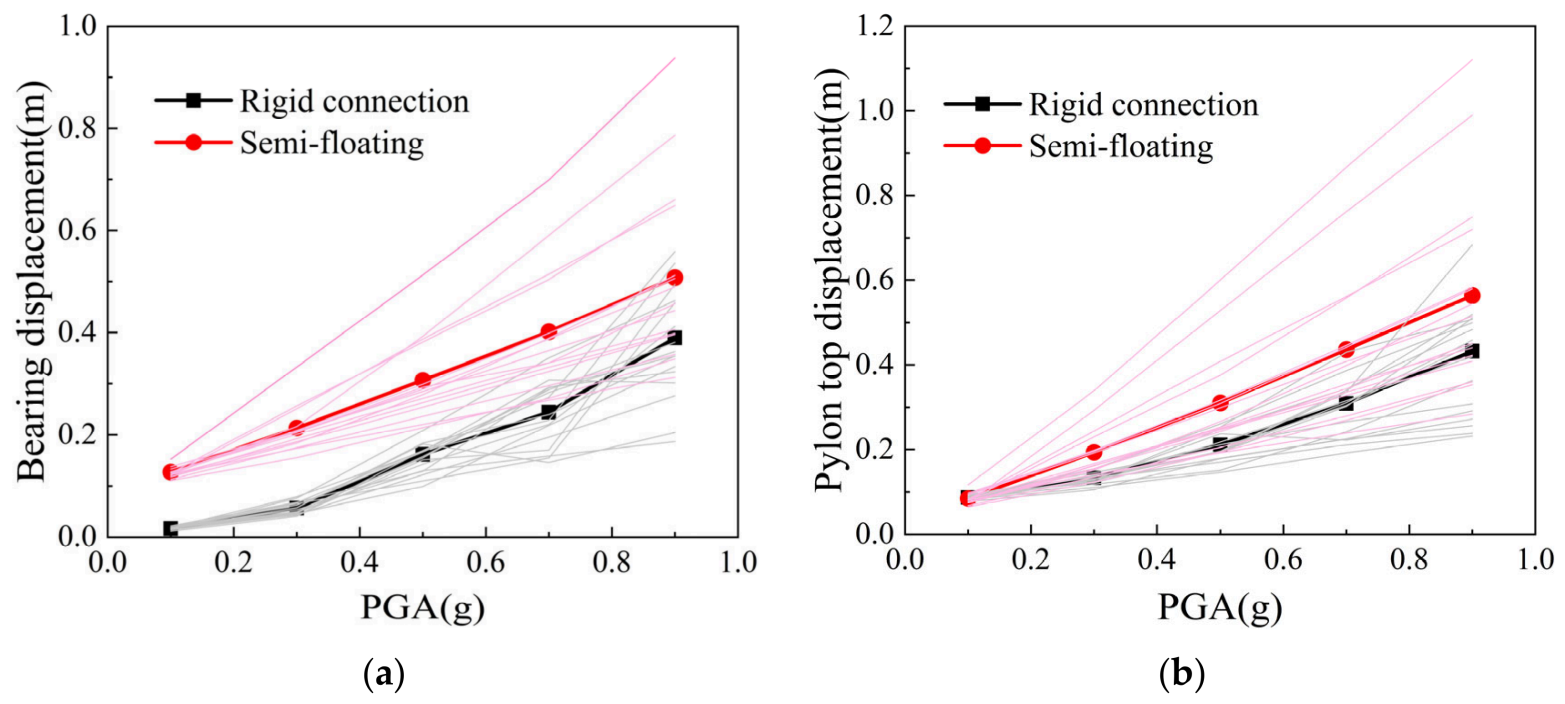

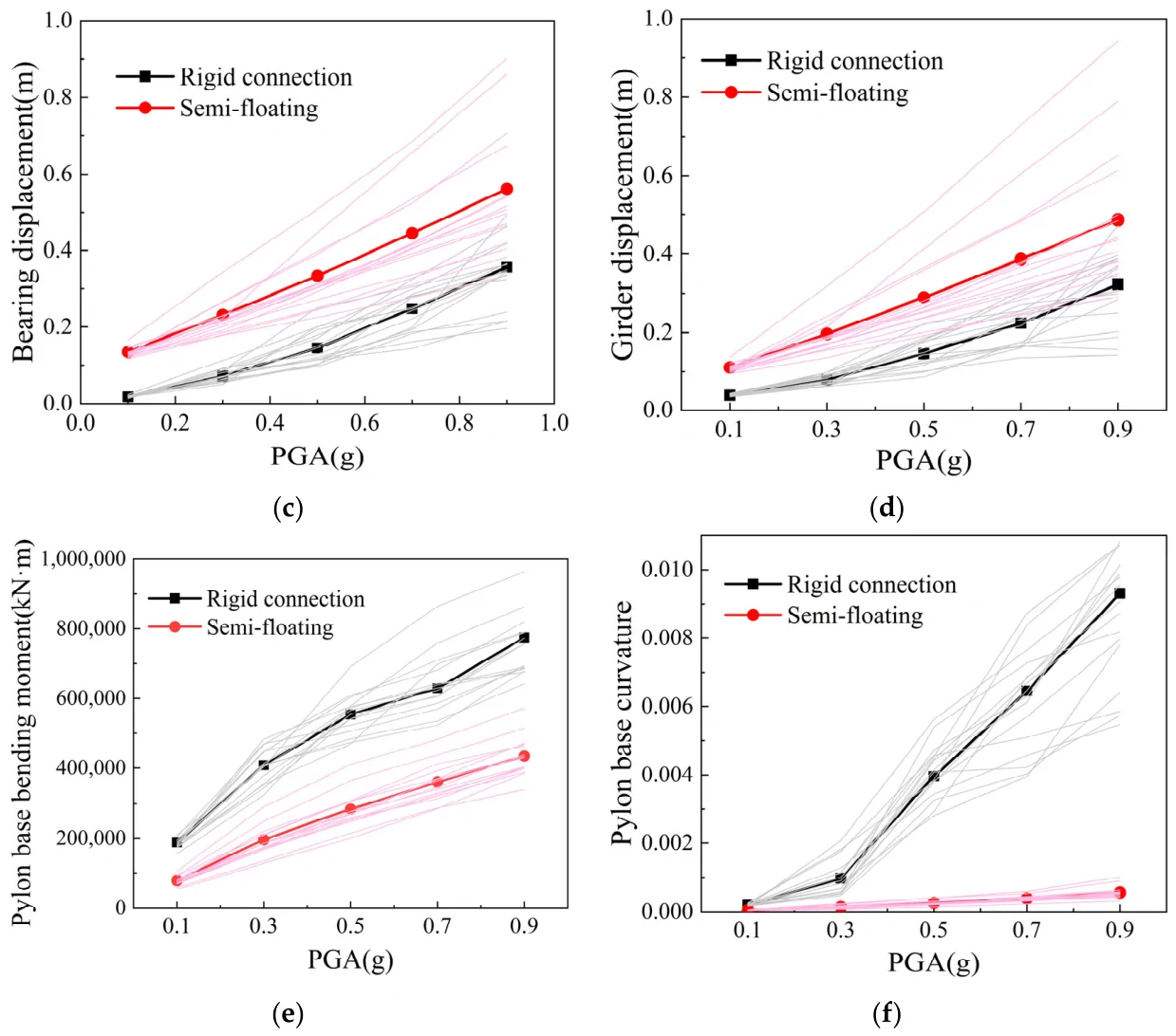
Comparison of seismic response of the rigidly connected and semi-floating systems: (**a**) pylon top displacement; (**b**) auxiliary pier bearing displacement; (**c**) right transition pier bearing displacement; (**d**) girder displacement; (**e**) pylon base bending moment; (**f**) pylon base curvature.

To investigate the seismic performance of the bridge in its completed state under different structural systems [4], a semi-floating system was developed as a benchmark for comparison [23]. For the pylon–girder rigid-connection system, the connection at the zero-block segment was represented using nodal master–slave constraints. For the semi-floating system, the same connection was modeled with link elements to capture the nonlinear longitudinal sliding behavior of the bearings [24]. Since the present cable-stayed bridge features a fixed connection between the tower and the girder, its seismic responses resemble those of short-period continuous girder bridges. For this reason, peak ground acceleration (PGA) is selected as the intensity measure in this study. Synthesizing the research findings from Zhong et al. (2016) [25], Barnawi et al. (2014) [10], and Agrawal et al. (2012) [26], fixed pylon–girder configurations significantly enhance the global longitudinal stiffness of cable-stayed bridges and restrain the long-period vibration of the main girder, leading to overall seismic behavior comparable to short-period continuous girders; the damage of bearings and pylons is governed by acceleration demands. Under strong ground motions, structural stiffness degradation induces shifts in the equivalent fundamental period, which causes substantial dispersion in spectral acceleration S_a_ (T_1_). In contrast, PGA is insensitive to structural nonlinearity. It enables damage evaluation for all critical components of the bridge, aligns with the PGA-based seismic fortification criteria in design codes, and is therefore more suitable as the ground motion intensity measure for the fixed pylon–girder cable-stayed bridge in this work. Incremental dynamic analyses (IDA) were conducted for both models, with the peak ground acceleration (PGA) increased from 0.1 g to 0.9 g at an interval of 0.2 g, to compare the evolution of seismic responses [23,27]. Figure 5 compares the seismic response of the rigidly connected and semi-floating systems. The results show that all the seismic response quantities increase monotonically with PGA [7].

As shown in Figure 5a, the pylon-top displacements in the rigidly connected system are consistently smaller than those in the semi-floating system. At PGA = 0.7 g, the pylon-top displacement of the rigidly connected system is approximately 25% lower than that of the semi-floating system. This difference arises from the distinct force-transfer mechanisms of the two systems. In the rigidly connected system, the girder inertial force is transmitted primarily to the pylon base through the rigid pylon–girder connection. In contrast, in the semi-floating system, a larger portion of the girder inertial force is transferred through the stay cables and applied to the pylon top, which significantly amplifies the pylon-top displacement [28].

Since the bearing-displacement responses at the left and right transition piers exhibit similar trends, only the right transition pier is discussed. Figure 5b–d show that the auxiliary-pier bearing displacement, right transition-pier bearing displacement, and girder displacement in the rigidly connected system are smaller than those in the semi-floating system [4]. At PGA = 0.7 g, the reductions are approximately 31%, 41%, and 38%, respectively. These differences are primarily due to the rigid pylon–girder connection, which provides strong longitudinal restraint on the girder in the rigidly connected system. In comparison, the semi-floating system relies on longitudinally frictional force of sliding bearings, which offers weaker restraint [23].

Figure 5e,f further indicate that the pylon-base bending moment and curvature in the rigidly connected system are greater than those in the semi-floating system. At PGA = 0.7 g, the pylon-base bending moment in the semi-floating system is about 46% lower than that of the rigidly connected system. In the rigidly connected system, the rigid connection compels the pylon to resist most of the girder inertial force, resulting in larger base demands. In the semi-floating system, longitudinal girder drift effectively lengthens the structural period and promotes energy dissipation, thereby reducing seismic demand at the pylon base [23]. This interpretation is consistent with the modal analysis results: the fundamental longitudinal periods of the rigidly connected system and the semi-floating system are 0.81 s and 2.805 s, respectively, while their fundamental transverse periods are 2.295 s and 2.174 s [28]. Meanwhile, the overall trend of seismic analysis results in this study is consistent with those reported by Han et al. regarding single-pylon cable-stayed bridges adopting semi-floating and fixed pylon–girder systems [29].

## 6. Seismic Response of Bridge in Construction Stages

**Figure 6:**
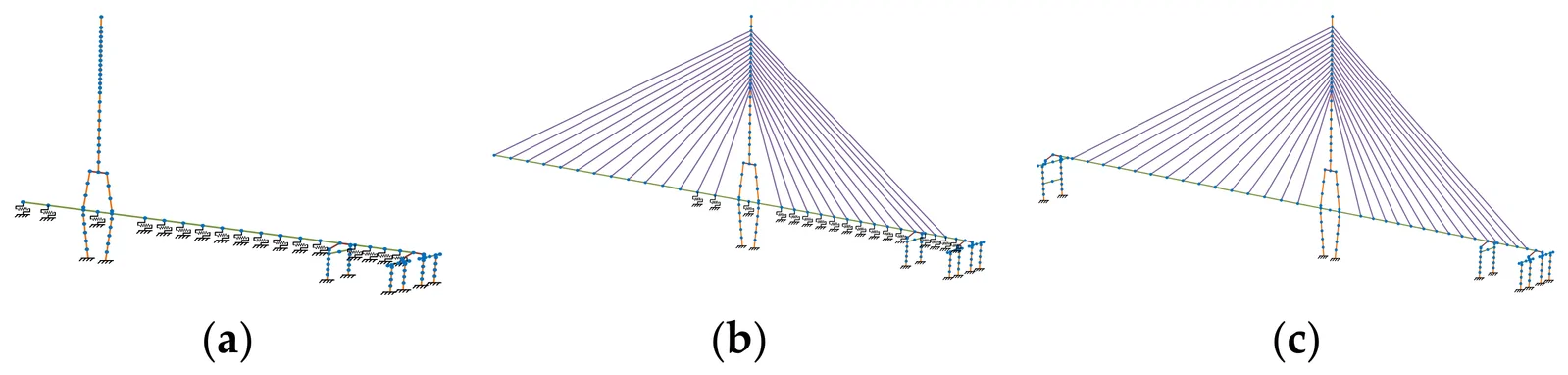
Finite element models for different construction stages: (**a**) pylon stage; (**b**) large cantilever stage; (**c**) completed bridge stage.

To accurately represent the structural characteristics during construction, two finite element models were developed corresponding to key construction phases: pylon construction and large cantilever [30]. The two phases incorporate temporary supports for the girder, modeled using friction elements where the horizontal friction force is dependent on the vertical pressure. A schematic of these construction stages is provided in Figure 6.

**Figure 7:**
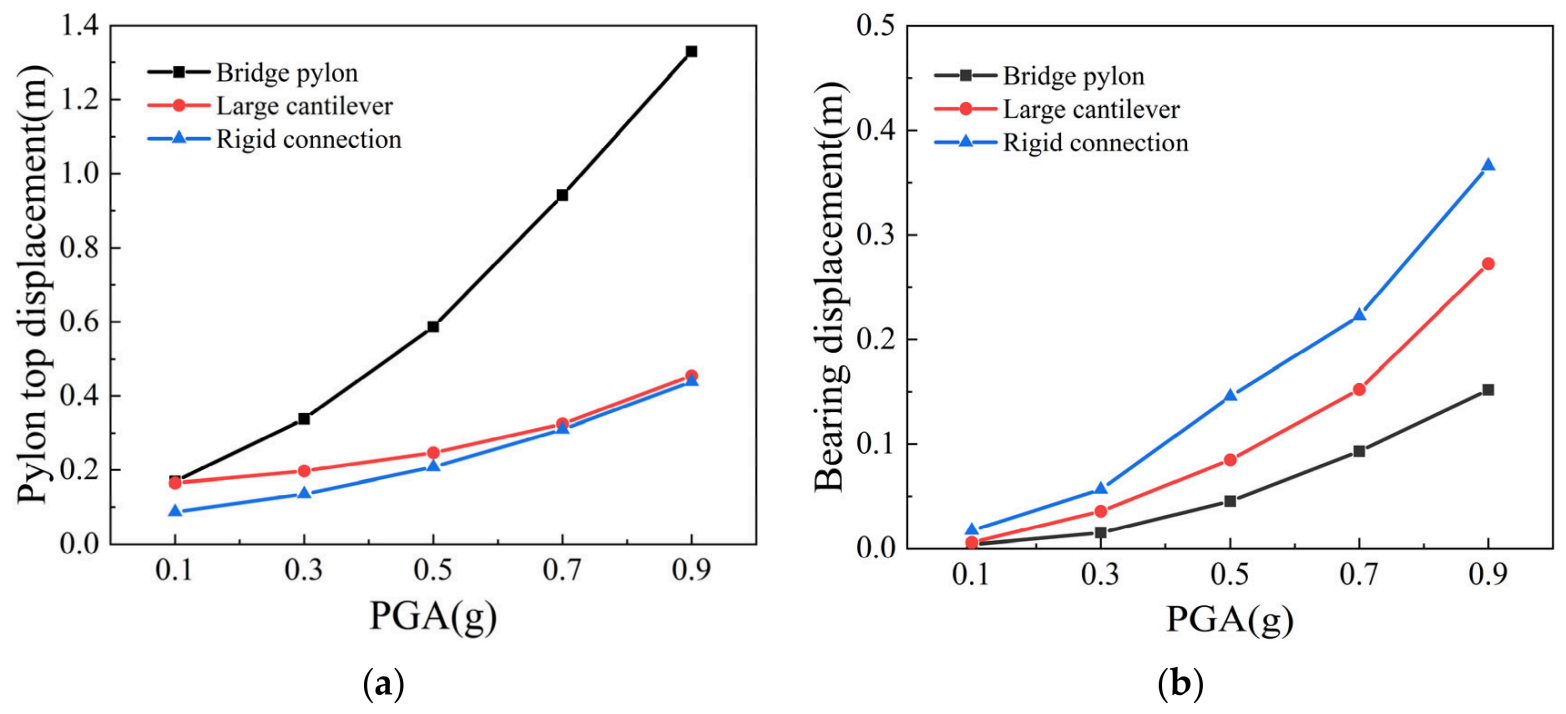

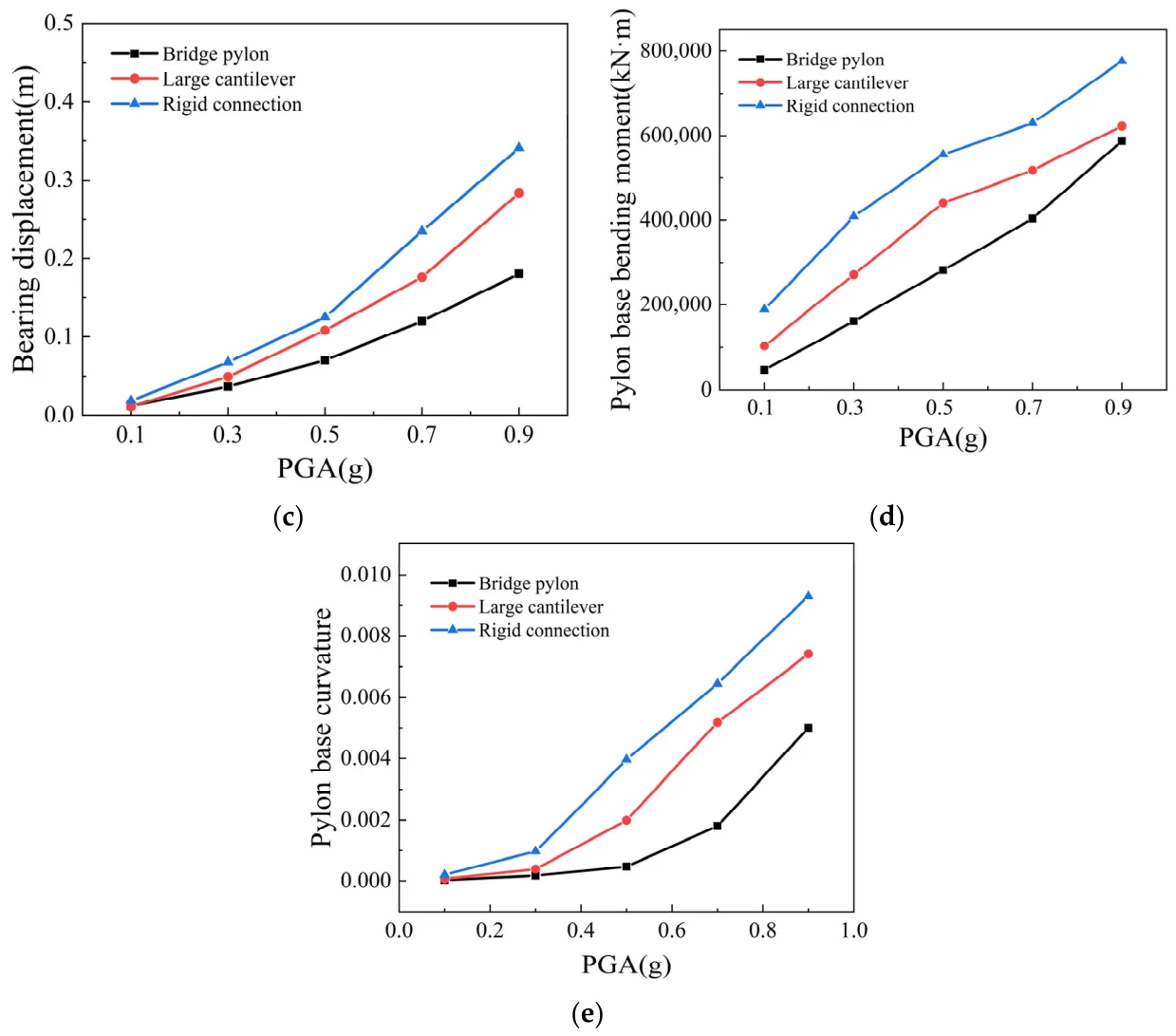
Seismic responses of the cable-stayed bridge for different construction stages: (**a**) pylon top displacement; (**b**) auxiliary pier bearing displacement; (**c**) right transition pier bearing displacement; (**d**) pylon base bending moment; (**e**) pylon base curvature.

Incremental dynamic analysis (IDA) was performed on three numerical models representing distinct construction stages. Figure 7 compares the seismic responses of the pylon–girder rigid-connection system under these construction stages [7]. Note that all the above-presented calculation results are the mean seismic response of a bridge subjected to 20 ground-motion records.

As shown in Figure 7a, at PGA = 0.7 g, the pylon-top displacements at the large cantilever stage and the completed-bridge stage are close to each other, and both are approximately 64% lower than that at the pylon stage. This indicates that the stay cables play an important role in restraining pylon-top displacement. Figure 7b,c show that the bearing displacements at both the auxiliary pier and the right transition pier increase progressively as construction advances. At a PGA of 0.7 g, the bearing displacement at the auxiliary pier in the large cantilever stage is 79% higher than that in the pylon stage, while the corresponding increase at the right transition pier reaches 57%. In the completed-bridge stage, these increases further rise to 141% and 90%, respectively, relative to the pylon stage. As illustrated in Figure 5e and Figure 7d, the pylon-base bending moment and curvature also increase with construction progression. At the same PGA level, compared with the pylon stage, the large cantilever stage shows increases of 7% in pylon-base bending moment and 50% in curvature, whereas the completed-bridge stage exhibits larger increases of 34% and 101%, respectively [31].

From the pylon stage to the large cantilever stage, the substantial increase in girder mass leads to larger seismic inertial forces, resulting in greater bearing displacements and larger internal forces at the pylon base. In the completed-bridge stage, the responses increase further because the removal of temporary supports reduces the longitudinal restraint of the girder, while the addition of secondary dead loads and counterweights further amplifies the girder inertial force. The above laws of displacement and internal force responses of the bridge at various construction stages show a consistent trend with the research results of long-span cable-stayed bridges presented by Liu et al. [32].

## 7. Parametric Analysis of Viscous Dampers

Viscous dampers are widely used for seismic control because they enhance energy dissipation through the velocity-dependent hysteretic behavior of viscous fluid [10], while having little influence on the structural period or stiffness [30]. The damping force is generally expressed as follows:

(3) $$F=Cv^{\alpha }$$

In the above equation, *F* denotes the damping force, *C* is the damping coefficient, v represents the relative velocity between the girder and pylon, and *α* is the velocity exponent, where *α* = 0.3 is adopted in this study [33,34].

This section examines the influence of the damping coefficient *C* on the seismic responses of both the rigidly connected system and the semi-floating system. Because temporary pylon–girder fixation is required for the semi-floating system during construction, the two systems are not distinguished in the numerical modeling of the pylon stage and the large cantilever stage [35].

**Figure 8:**
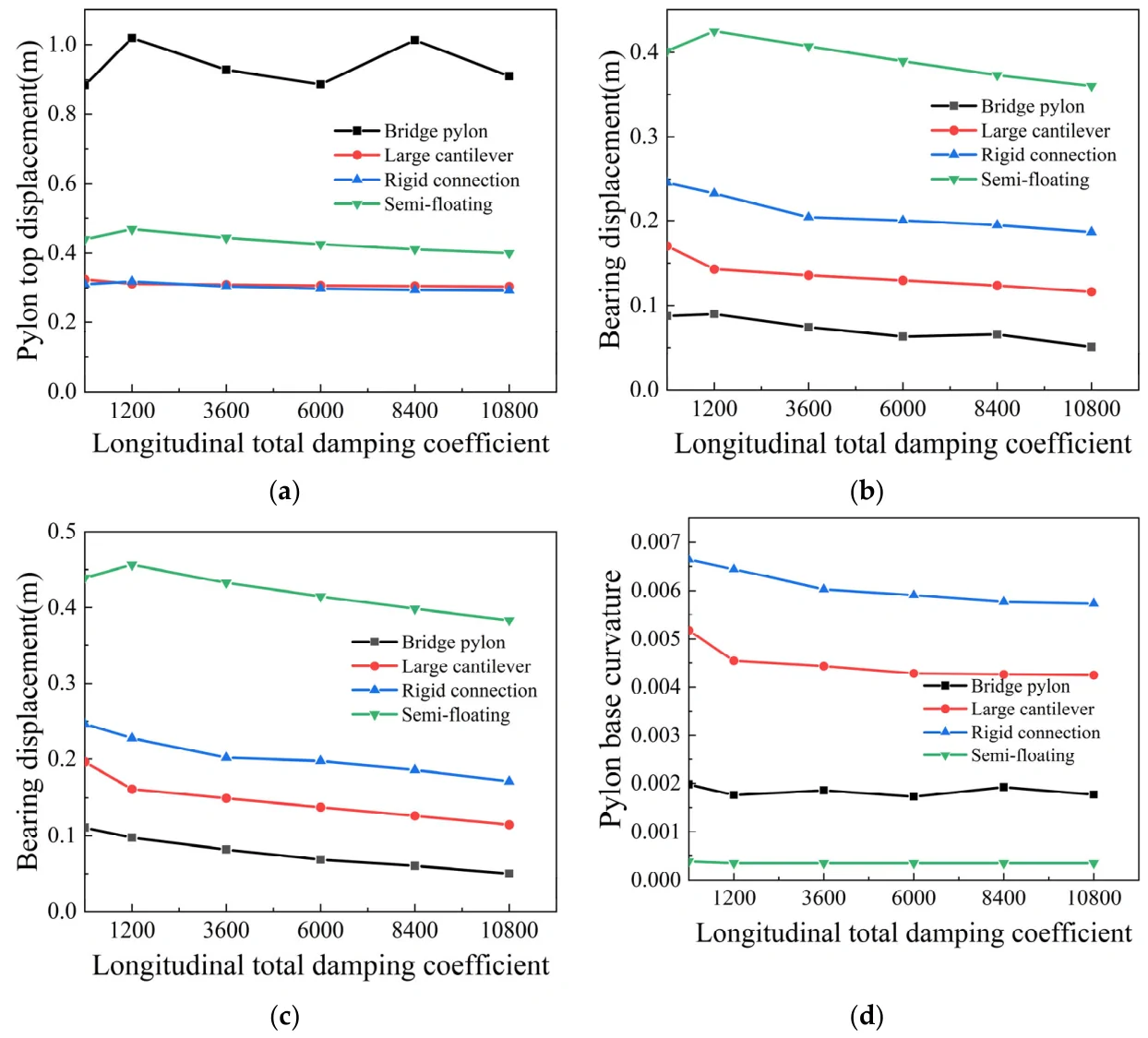
Seismic responses of the cable-stayed bridge with different viscous damping coefficients: (**a**) pylon top displacement; (**b**) auxiliary pier bearing displacement; (**c**) right transition pier bearing displacement; (**d**) pylon base curvature.

Dampers are arranged at all bearing locations. To ensure the same total longitudinal damping force in the two structural systems, the total longitudinal damping coefficient is varied from 1200 to 10,800 kN·(s/m)^0.3^, with an increment of 2400 kN·(s/m)^0.3^. Dynamic analyses are carried out under a PGA of 0.7 g [36]. Figure 8 presents the parametric study results for the rigidly connected system and the semi-floating system. Two typical construction stages, i.e., the pylon-completed stage and the large-cantilever stage, are considered in the analysis. The case with a zero damping coefficient corresponds to the structural condition without viscous dampers. Note that all the above-mentioned calculation results represent the mean seismic response of a bridge subjected to 20 ground-motion records.

The results show that viscous dampers are effective in reducing bearing displacements, whereas their influence on pylon-top displacement and curvature is limited. As shown in Figure 8b,c, the bearing displacements decrease progressively as the damping coefficient increases. When the total longitudinal damping coefficient reaches 10,800 kN·(s/m)^0.3^, the bearing displacements at the completed-bridge stage are reduced by about 24% in the rigidly connected system and about 10% in the semi-floating system, relative to the corresponding cases without dampers. By contrast, Figure 8a,d indicate only slight reductions in pylon-top displacement and pylon-base curvature [10]. This trend agrees well with the findings on the application of viscous dampers in bridges reported by Ruan et al. [37].

## 8. Fragility Analysis

### 8.1. Principles of Fragility Analysis

Seismic fragility refers to the probability that a structure reaches or exceeds a specified damage state under earthquakes of different intensities. The fragility function is expressed as Equation (4) [38]:

(4) $$F=P(LS\left|IM=y)\right.$$

where *LS* denotes a specific damage state of a component or system, and *IM* is the ground-motion intensity measure, which in this study specifically refers to peak ground acceleration (PGA).

Cornell et al. [39] proposed that the relationship between seismic demand and ground-motion intensity can be fitted by Equation (5):

(5) $$S_{d}/S_{c}=aIM^{b}$$

where *a* and *b* are unknown coefficients determined by the least-squares method; *S_d_* is the seismic demand of the vulnerable bridge components; and *S_c_* is the seismic capacity of the vulnerable bridge components corresponding to different damage limit states.

Taking the natural logarithm of both sides of Equation (6) yields

(6) $$\ln(S_{d}/S_{c})=\ln\left(a\right)+b\ln\left(IM\right)$$

The dispersion of seismic demand can be calculated by Equation (7):

(7) $$\sigma =\sqrt{\frac{\sum_{i=1}^{N}{\left[\ln(S_{d}/S_{c})-\ln(aIM^{b})\right]}^{2}}{N-2}}$$

where *N* is the total number of seismic analysis cases.

Accordingly, the conditional probability that a vulnerable bridge component exceeds a specified damage state under a given intensity measure *IM* is calculated as

(8) $$P_{f}=P[\frac{S_{d}}{S_{c}}\geq 1]=1-\Phi \left(\frac{\ln(1)-\ln(S_{d}/S_{c})}{\sigma }\right)=\Phi (\frac{\ln(S_{d}/S_{c})}{\sigma })=\Phi (\frac{\ln(aIM^{b})}{\sigma })$$

where Φ is the cumulative distribution function of the standard normal distribution.

In addition to component fragility, system fragility is required to evaluate the overall seismic vulnerability of the bridge, because damage to any critical component may impair the structural performance or even trigger system-level failure. Unlike component fragility, system fragility accounts for the combined effects of multiple vulnerable components and their statistical correlations under the same ground-motion intensity. In this study, the system fragility is established based on the joint probabilistic seismic demand models (JPSDMs) of key components together with their capacity models. A Monte Carlo simulation procedure is then employed to generate a large number of demand samples and to estimate the probability that the bridge system reaches or exceeds a specified damage state [38].

### 8.2. Damage Limit States

As the primary lateral-force-resisting members of a bridge subjected to seismic excitation, pylons and piers play a critical role in seismic response assessment [40]. Owing to their geometric characteristics and load-carrying behavior, various damage indices have been adopted in previous studies to define their damage limit states, among which the most commonly used are pier/pylon top displacement and curvature. In the present study, curvature is selected as the damage index for both pylons and piers. The corresponding curvature thresholds are classified into four damage levels: slight, moderate, extensive, and complete. Slight damage is deemed to occur when the outermost reinforcing steel first yields, and the corresponding curvature is denoted as *φ*_1_. Moderate damage is defined as the onset of spalling of the cover concrete, corresponding to *φ*_2_. Extensive damage is taken to occur when the core concrete reaches its peak compressive stress, corresponding to *φ*_3_. Complete damage is defined when the core concrete reaches its ultimate compressive strain, corresponding to *φ*_4_. Detailed values are listed in Table 2.

Under seismic excitation, bearings are critical components in the bridge load-transfer system, and their damage has a direct influence on the overall seismic performance of the structure [4]. In this study, the damage state of the bearings is assessed in terms of the relative displacement between the girder and the cap beam. Complete damage is defined when the bearing displacement reaches the maximum seating length of 1.8 m at the girder end relative to the cap beam, corresponding to the onset of superstructure unseating failure. The limits for slight, moderate, and extensive damage are specified as 0.1, 0.3, and 0.75 times the complete-damage threshold, respectively.

### 8.3. Component Fragility Analysis

In this study, incremental dynamic analysis (IDA) was employed, with PGA scaled from 0.1 g to 1.9 g at an increment of 0.2 g, to evaluate the fragility of the pylon base, the base of the right transition pier [31], and the bearing at the right transition pier during different construction stages [41]. For the completed-bridge stage, both the rigidly connected system and the semi-floating system were considered. However, because temporary pylon–girder fixation is required for the semi-floating system during construction, no distinction is made between the two structural systems in the first two construction stages.

**Figure 9:**
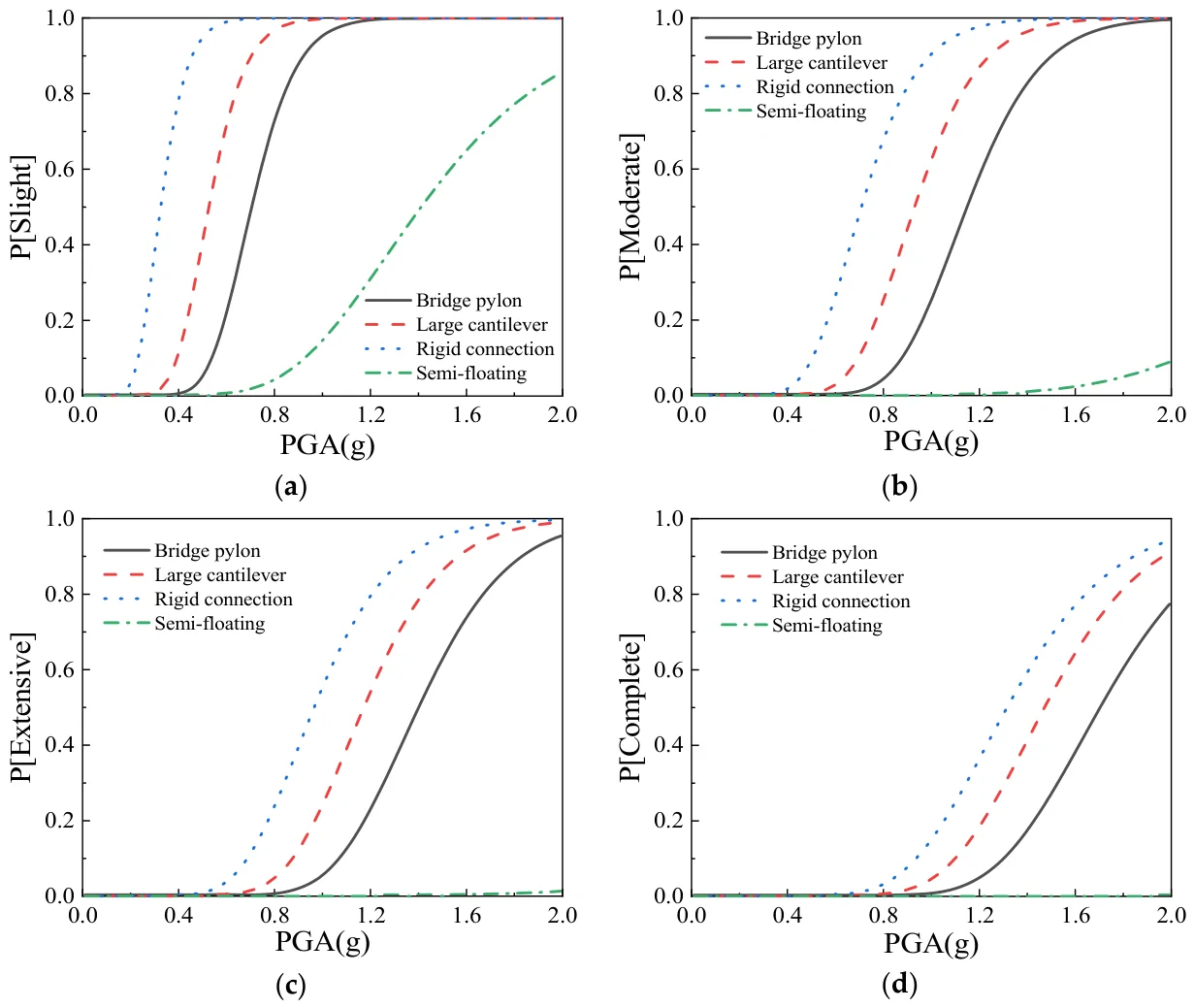
Fragility curves of the pylon base: (**a**) slight damage; (**b**) moderate damage; (**c**) extensive damage; (**d**) complete damage.

Figure 9 presents the seismic fragility curves of the pylon-base section under different damage states. At a PGA of 0.68 g, the damage probabilities of the pylon base in the pylon stage, the large cantilever stage, and the completed rigidly connected system stage for a slight damage limit state are 42%, 86%, and 99%, respectively, indicating that the damage probability increases progressively with construction progress. This trend can be attributed to the increase in girder mass during construction, which results in larger seismic inertial forces. The damage probability of the pylon-base section in the semi-floating system at the completed-bridge stage is significantly lower than that in the rigidly connected system. This is because some of the longitudinal seismic inertial force of the girder in the semi-floating system is dissipated through the longitudinal sliding bearings between the girder and the pylon, while the remainder is transmitted to the pylon through the stay cables. Moreover, below the bifurcation of the two pylon legs, the bending moment in the pylon is converted into differential axial forces of the two legs, thereby reducing flexural damage at the pylon base. By contrast, in the rigidly connected system, the girder inertial force is transmitted directly from the pylon–girder connection to the pylon base owing to the rigid connection, resulting in more severe flexural damage [42].

**Figure 10:**
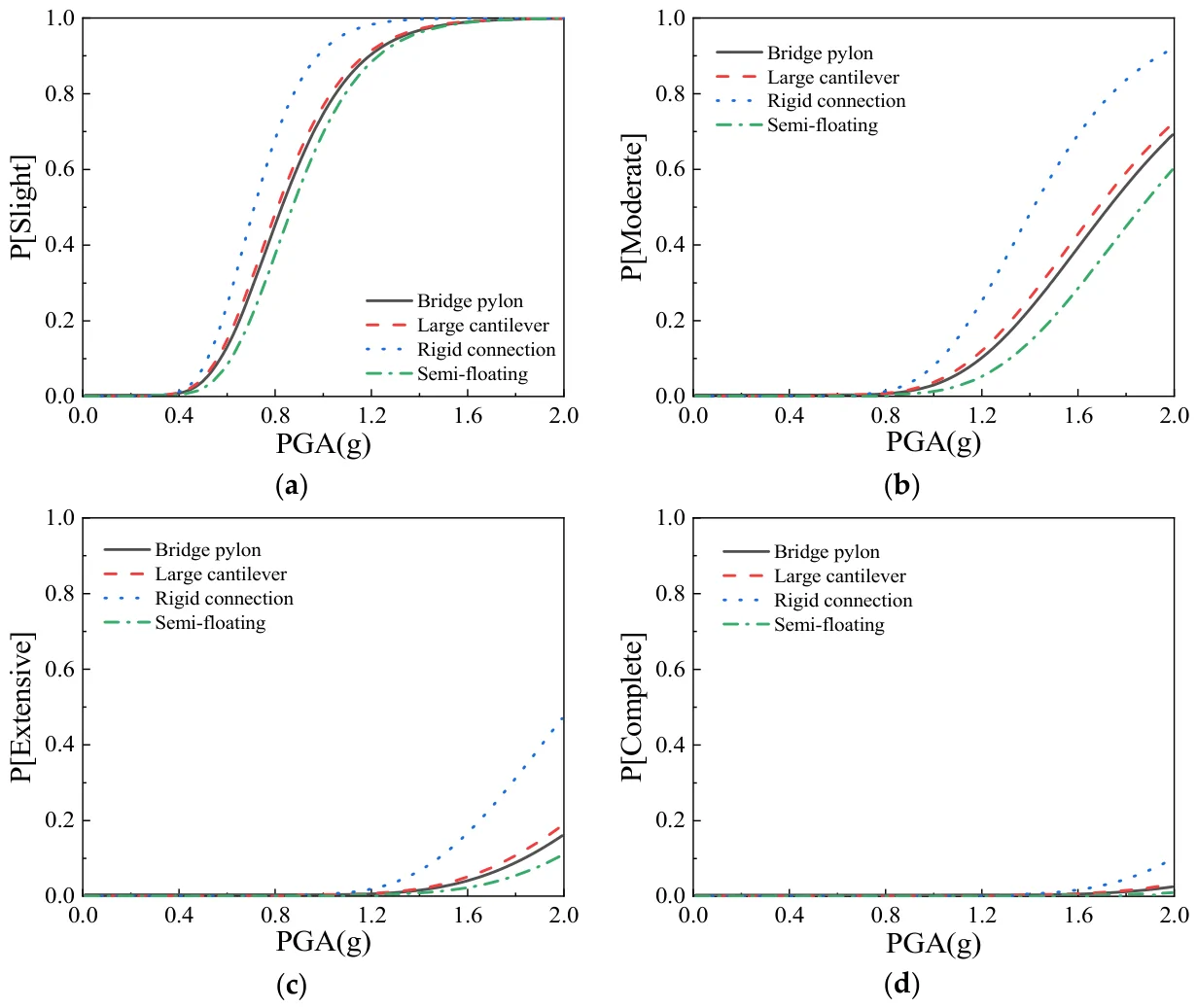
Fragility curves of the base of the right transition pier: (**a**) slight damage; (**b**) moderate damage; (**c**) extensive damage; (**d**) complete damage.

Figure 10 presents the seismic fragility curves of the base of the right transition pier under different damage states. As shown in the figure, the damage probability at the pier base is higher for the rigidly connected system at the completed-bridge stage, whereas it is lower for the semi-floating system at the completed-bridge stage. At a PGA of 0.68 g, the probabilities of slight damage in the pylon stage, the large cantilever stage, the completed rigidly connected system, and the completed semi-floating system are 24%, 26%, 41%, and 17%, respectively. For the rigidly connected system, the damage probability increases progressively from the pylon stage to the large cantilever stage and then to the completed-bridge stage. This can be attributed to two factors. First, the temporary supports of side-span girder are removed at the completed-bridge stage. Second, as construction progresses, the girder mass increases. Together, these effects lead to increases in the axial force in the transition pier and the friction force at the pier top.

**Figure 11:**
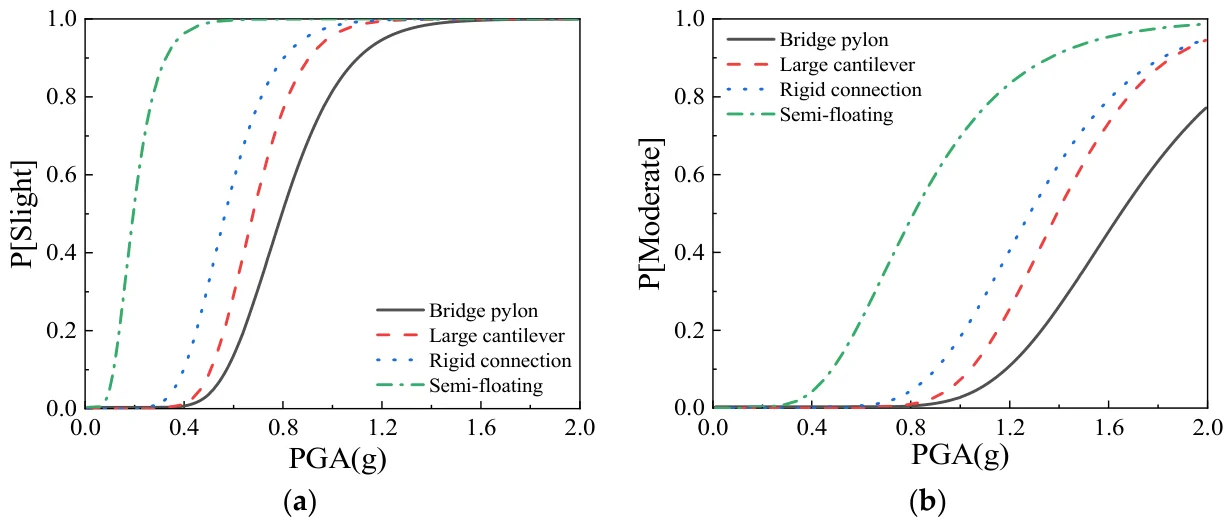

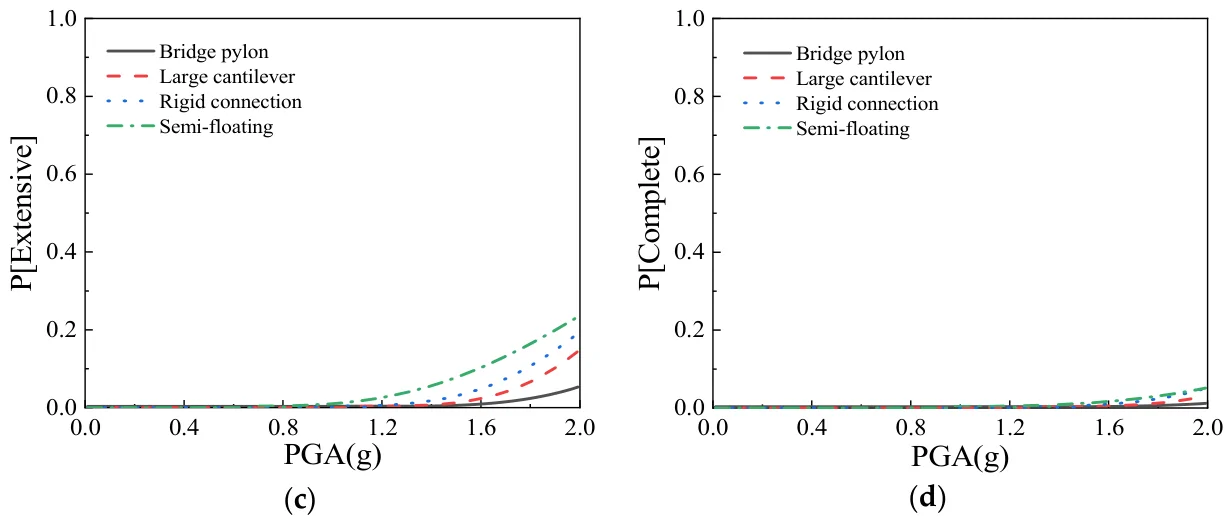
Fragility curves of the bearing at the right transition pier: (**a**) slight damage; (**b**) moderate damage; (**c**) extensive damage; (**d**) complete damage.

Figure 11 presents the seismic fragility curves of the bearing at the right transition pier under different damage states. As shown in the figure, the probability of bearing damage is higher in the semi-floating system at the completed-bridge stage, whereas it is lower in the rigidly connected system. Specifically, at a PGA of 0.68 g, the probabilities of slight damage in the pylon stage, the large cantilever stage, the completed rigidly connected system, and the completed semi-floating system are 26%, 49%, 74%, and 99%, respectively. This can be attributed to the fact that, in the semi-floating system, the girder is supported by sliding bearings at the piers, which provide weaker restraint to longitudinal movement of the girder and thus allow larger relative longitudinal displacements under seismic excitation.

### 8.4. System Fragility Analysis

The system-level seismic fragility curves were generated within a Monte Carlo framework based on the JPSDM model to compare the overall vulnerability of the bridge [43].

**Figure 12:**
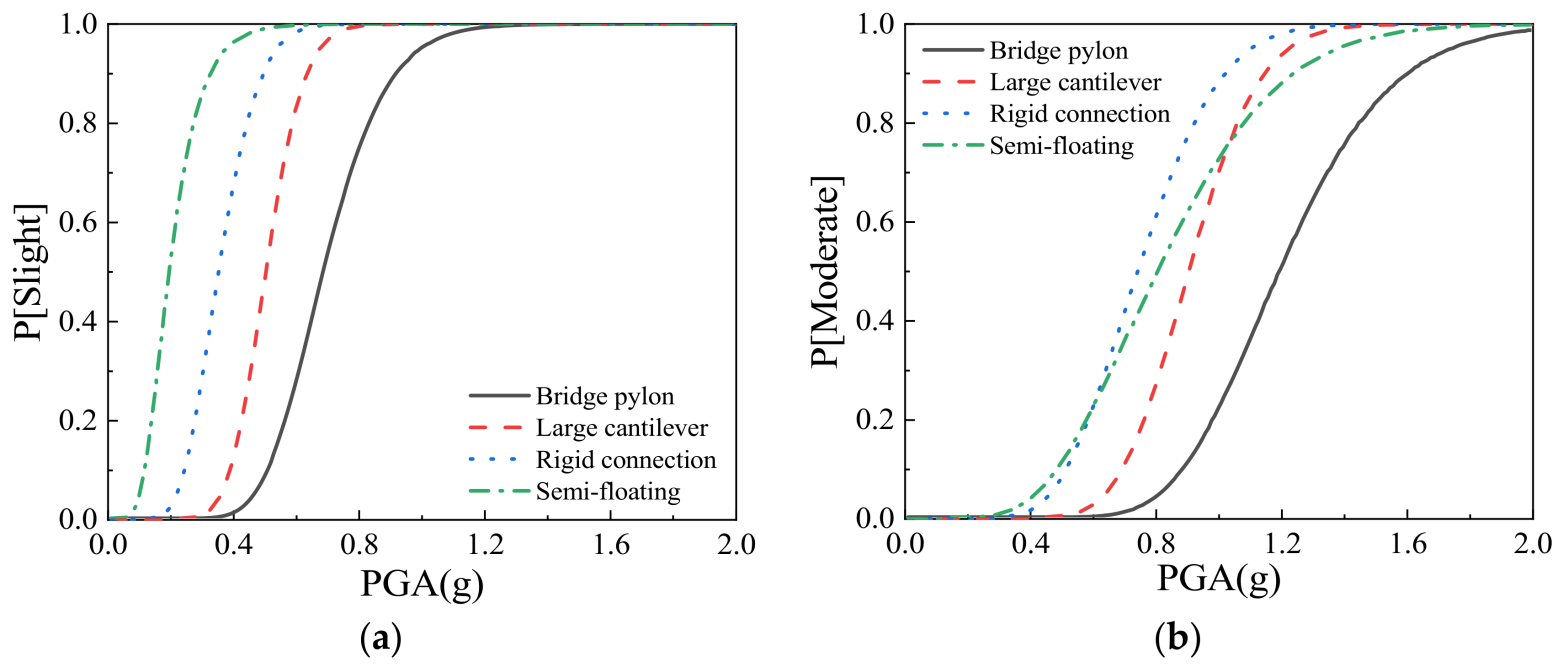

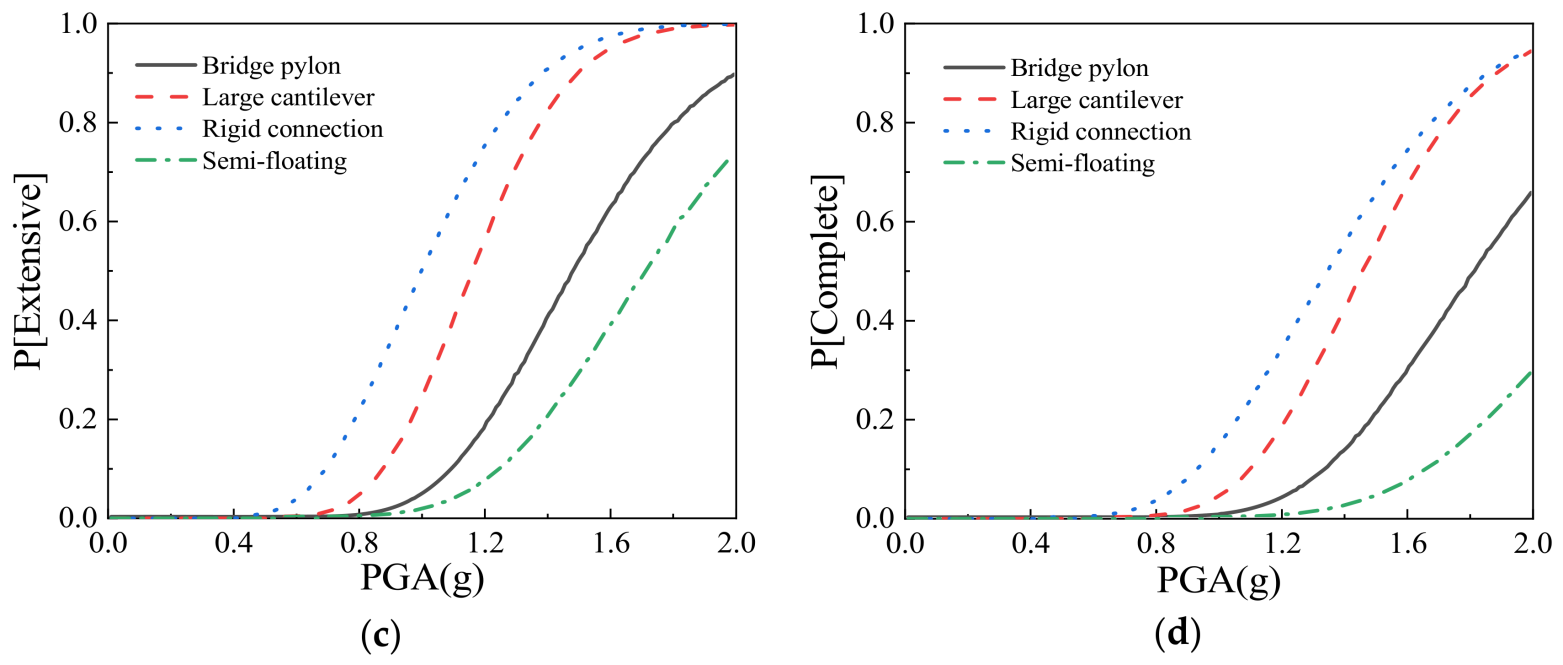
System-level fragility curves: (**a**) slight damage; (**b**) moderate damage; (**c**) extensive damage; (**d**) complete damage.

Figure 12 presents the system-level seismic fragility curves for different construction stages. As shown in Figure 12a, for slight damage, the damage probabilities in descending order are those of the completed semi-floating system, the completed rigidly connected system, the large cantilever stage, and the pylon stage. Specifically, at a PGA of 0.68 g, the probabilities of slight damage in the pylon stage, the large cantilever stage, the completed rigidly connected system, and the completed semi-floating system are 48%, 95%, 99%, and 99%, respectively. The order of slight damage probabilities for different components is consistent with that in Figure 11a, indicating that the slight damage probability is governed by the damage probability of bearings on transition piers. For extensive and complete damage, the damage probabilities of the semi-floating system are lower than those of the rigidly connected system. The ranking of extensive and complete damage probabilities among different components is consistent with that shown in Figure 9c,d, which indicates that the probabilities of extensive and complete damage are governed by the pylon-base curvature.

## 9. Conclusions

A single-pylon cable-stayed bridge was selected as the prototype, and nonlinear finite-element models were established to investigate the seismic response and fragility of different structural systems and construction stages. The main conclusions are as follows:

(1) For the completed bridge, the pylon–girder rigid-connection system provides stronger longitudinal restraint than the semi-floating system. Consequently, the pylon-top displacement, the bearing displacements at the auxiliary pier and the right transition pier, and the girder displacement are smaller. However, this configuration transfers more seismic inertial force to the pylon base, leading to larger bending moments and curvature at the pylon base.
(2) The pylon-top displacement reaches its maximum during the pylon construction stage, due to the absence of restraint from stay cables. From the pylon stage to the large cantilever stage and then to the completed-bridge stage, the bearing displacements and the seismic demand at the pylon base increase continuously.
(3) The parametric analysis shows that increasing the total longitudinal damping coefficient can reduce the bearing displacements at the auxiliary pier and the right transition pier. When the total longitudinal damping coefficient reaches 10,800 kN·(s/m)^0.3^, the bearing displacement at the completed-bridge stage is reduced by about 20% in the rigid-connection system, compared with the undamped case.
(4) The two structural systems exhibit different vulnerability characteristics. In the completed rigid-connection system, the pylon base and the base of the right transition pier are more vulnerable, whereas in the completed semi-floating system, the bearing at the right transition pier shows a higher damage probability.
(5) In terms of system fragility, the damage probability of the rigid connection system gradually rises as the construction stage evolves from the pylon stage to the large-cantilever stage, and finally to the completed bridge. The probability of slight damage for the semi-floating system is higher than that of the rigidly connected system, whereas the semi-floating system exhibits lower probabilities of extensive and complete damage compared with the rigidly connected system.

The seismic fragility analysis method for cable-stayed bridges with multiple construction stages and structural systems proposed in this paper exhibits prominent engineering applicability. At the bridge design stage, this method can quantitatively evaluate the damage evolution of all bridge components under various structural schemes and damper layout parameters, providing quantitative references for system optimization and parameter design of longitudinal viscous dampers. For maintenance and retrofitting of existing cable-stayed bridges, the structural and component fragility curves can be adopted to identify seismic vulnerable elements, and the priority of component repair and seismic retrofitting can be determined according to the exceedance probabilities of different damage states.

The conclusions drawn in this study are only applicable to the prototype cable-stayed bridge subjected to longitudinal seismic excitation. The numerical model adopts simplified assumptions of fixed foundations and an elastic main girder, with constant damage threshold values specified. In addition, the ground motion records selected herein span a limited parameter range. Accordingly, the presented findings cannot be directly generalized to other loading scenarios or distinct bridge configurations.

## Figures and Tables

**Table 1:** Properties of input ground motions.

No.	Earthquake	Year	Station	Magnitude	Epicentral Distance (km)	PGA (g)
1	Kern County	1952	Taft Lincoln School	7.36	38.42	0.159
2	Northern Calif-03	1954	Ferndale City Hall	6.5	26.72	0.163
3	Borrego Mtn	1968	El Centro Array #9	6.63	45.12	0.133
4	San Fernando	1971	Lake Hughes #1	6.61	22.23	0.151
5	San Fernando	1971	Palmdale Fire Station	6.61	24.16	0.112
6	San Fernando	1971	Pasadena-CIT Athenaeum	6.61	25.47	0.097
7	Tabas Iran	1978	Boshrooyeh	7.35	24.07	0.106
8	Imperial Valley-06	1979	Delta	6.53	22.03	0.236
9	Imperial Valley-06	1979	El Centro Array #13	6.53	21.98	0.118
10	Irpinia Italy-02	1980	Rionero In Vulture	6.2	22.68	0.100
11	Coalinga-01	1983	Cantua Creek School	6.36	23.78	0.225
12	Coalinga-01	1983	Parkfield-Cholame 2WA	6.36	43.83	0.110
13	Coalinga-01	1983	Parkfield-Cholame 3W	6.36	44.82	0.098
14	Hollister-01	1961	Hollister City Hall	5.6	19.55	0.059
15	Point Mugu	1973	Port Hueneme	5.65	15.48	0.128
16	Livermore-01	1980	San Ramon—Eastman Kodak	5.8	15.19	0.149
17	Victoria_ Mexico	1980	Chihuahua	6.33	18.53	0.151
18	Chalfant Valley-01	1986	Bishop—LADWP South St	5.77	23.38	0.126
19	Chalfant Valley-02	1986	Long Valley Dam (L Abut)	6.19	18.3	0.082
20	Morgan Hill	1984	Hollister Differential Array #3	6.19	26.42	0.079

**Table 2:** Damage limit states of piers and pylon (×10^−2^ m^−1^).

Location	Stage	*φ*_1_	*φ*_2_	*φ*_3_	*φ*_4_
Pylon base	Pylon stage	0.1871	0.5663	0.9012	1.3908
	Large cantilever stage	0.1747	0.5428	0.8594	1.3526
	Completed-bridge stage	0.1581	0.5099	0.8126	1.2944
Base of right transition pier	Pylon stage	0.4090	1.2175	2.2933	3.5008
	Large cantilever stage	0.4090	1.2175	2.2933	3.5008
	Completed-bridge stage	0.4226	1.2495	2.2133	3.5550

## Data Availability

The original contributions presented in this study are included in the article. Further inquiries can be directed to the corresponding author.
